# Large language models for structured cardiovascular data extraction: a foundation for scalable research and clinical applications

**DOI:** 10.1093/ehjdh/ztaf127

**Published:** 2025-11-14

**Authors:** Wouter van der Loo, Viktor van der Valk, Tim van den Broek, Douwe Atsma, Marius Staring, Roderick Scherptong

**Affiliations:** Department of Cardiology, Leiden University Medical Center, Leiden, The Netherlands; Department of Radiology, Leiden University Medical Center, Leiden, The Netherlands; Netherlands Organisation for Applied Scientific Research, Leiden, The Netherlands; Department of Cardiology, Leiden University Medical Center, Leiden, The Netherlands; Department of Radiology, Leiden University Medical Center, Leiden, The Netherlands; Department of Cardiology, Leiden University Medical Center, Leiden, The Netherlands

**Keywords:** Artificial intelligence, Large language models, Acute coronary syndrome, Electronic health records, Cardiac imaging reports, Natural language processing

## Abstract

**Aims:**

Automated extraction of information from cardiac reports would benefit both clinical reporting and research. Large language models (LLMs) hold promise for such automation, but their clinical performance and practical implementation across various computational environments remain unclear. This study aims to evaluate the feasibility and performance of LLM-based classification of echocardiogram and invasive coronary angiography reports, using real-world clinical data across local, high-performance computing and cloud-based platforms.

**Methods and results:**

The angiography and echocardiography reports of 1000 patients, admitted with acute coronary syndrome, were labelled for multiple key diagnostic elements, including left ventricular function (LVF), culprit vessel, and acute occlusions. Report classification models were developed using LLMs via (i) prompt-based and (ii) fine-tuning approaches. Performance was assessed across different model types and compute infrastructures, with attention to class imbalance, ambiguous label annotations, and implementation costs. Large language models demonstrated strong performance in extracting structured diagnostic information from cardiac reports. Cloud-based models (such as GPT-4o) achieved the highest accuracy (0.87 for culprit vessel and 1.0 for LVF) and generalizability, but also smaller models run on a local high-performance cluster achieved reasonable accuracy, especially for less complex tasks (0.634 for culprit vessel and 0.984 for LVF). Classification was feasible with minimal pre-processing, enabling potential integration into electronic health record systems or research pipelines. Class imbalance, reflective of real-world prevalence, had a greater impact on fine-tuning approaches.

**Conclusion:**

Large language models can reliably classify structured cardiology reports across diverse computed infrastructures. Their accuracy and adaptability support their use in clinical and research settings, particularly for scalable report structuring and dataset generation.

## Graphical Abstract

**Graphical Abstract:**
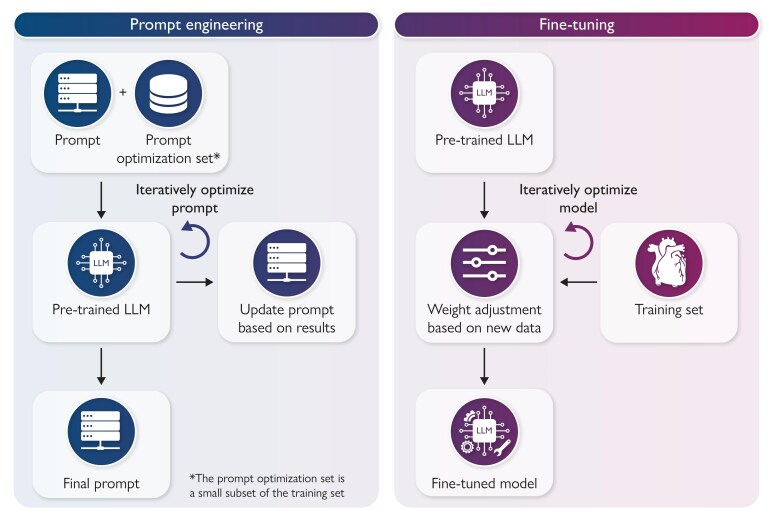

## Introduction

Invasive coronary angiography (ICA) and transthoracic echocardiography (TTE) are cornerstone imaging modalities in cardiology, essential for diagnosis, treatment, and follow-up in patients with coronary artery disease (CAD).^1,2^ Both procedures result in detailed reports, typically recorded in a semi-structured free-text format within electronic health records (EHRs). While this format enables flexible, context-rich documentation, standardization is limited, restricting its usability for data-driven research, clinical decision support systems, and artificial intelligence (AI) applications.^3–5^

The absence of structured labels presents a critical bottleneck in AI-driven research, where large, accurately annotated datasets are essential for the development of robust models.^6^ Manual annotation remains the predominant method for dataset creation, yet it is labour-intensive and prone to human error, making it impractical for large-scale supervised learning.^7^ As a result, much of the valuable data generated in clinical practice remains inaccessible.^8^

Recent advances in natural language processing (NLP), particularly the emergence of large language models (LLMs), offer new opportunities for automating the extraction of structured information from free-text reports.^9^ These models, built on transformer-based architectures with attention mechanisms, have demonstrated superior contextual understanding and scalability compared with earlier NLP techniques.^10^ Large language models have already shown promise in automating structured data extraction from free-text radiology reports.^6,11^

We hypothesize that LLM-based methods can automate the structured classification of ICA and TTE reports, enabling scalable dataset creation for AI applications and secondary data use. These methods also offer a robust foundation for extracting standardized data elements required for clinical registries, quality improvement programmes, and mandatory health system reporting, thereby reducing administrative burden, enhancing data completeness, and supporting data-driven oversight across diverse healthcare settings. In this study, two distinct LLM-based approaches, prompt engineering and fine-tuning, were developed and evaluated using free-text cardiology reports obtained from routine clinical practice. In prompt engineering, task instructions, optionally with several examples, guide a pre-trained model without changing its parameters. In fine-tuning, the model’s weights are updated based.

## Methods

### Datasets

A comprehensive pseudo-anonymized database was constructed from the EHRs of patients treated for acute coronary syndrome (ACS) at Leiden University Medical Centre (LUMC), The Netherlands, between 2010 and 2024. This database encompassed all clinical data generated during initial treatment and subsequent follow-up of these patients. The study protocol (nWMODIV2-2022006) was approved by the institutional review board of LUMC, which waived the requirement for obtaining informed consent for the use of data from individual patients. All procedures were conducted in accordance with institutional guidelines and regulations. From the source database, two study-specific datasets were created by random sampling: One consisting of 1000 ICA reports and the other comprising 1000 TTE reports. All reports were written in Dutch, adhered to a semi-structured format, and were composed by board-certified cardiologists during routine clinical care. None of the reports contained personally identifiable information, apart from clinical details specific to the modality. Only the textual content of the reports, without any accompanying metadata, was processed through the LLMs. Data processing and model inference were performed within secure, encrypted environments compliant with European data protection regulations, using only anonymized reports that underwent manual review for the presence of personally identifiable data prior to processing. Textual data were pre-processed to enhance quality and consistency prior to model training. Automated cleaning scripts based on regular expressions, a technique for identifying and modifying text patterns, were used to remove unnecessary spaces, redundant line breaks, and formatting artefacts introduced during data extraction. This process reduced noise, corrected structural inconsistencies, and standardized the text format across all reports, which were written and transferred to different data formats (finally to the XML standard) by different software packages used in the hospital over the years.

#### Data labelling

A structured annotation protocol was employed, involving two independent expert reviewers who each annotated all 2000 reports across two iterative rounds. Prior to the first round, detailed annotation guidelines were established and subsequently refined after each iteration to improve label consistency and annotation accuracy. Final labels were determined by resolving any disagreements through consensus discussions between the annotators.

To assess the difficulty of the labelling task and establish a baseline for evaluating the LLM performance, average manual annotation scores were calculated. These scores were derived by comparing each annotator’s final-round labels with the agreed-upon consensus labels.

Invasive coronary angiography reports were labelled for attributes relevant to diagnosis and treatment decisions: presence of acute occlusion, presence of bypass grafts, presence of significant epicardial CAD, treatment strategy [percutaneous coronary intervention (PCI), referral for coronary artery bypass grafting (CABG), or medical treatment only], and identification of culprit vessel(s), with labels assigned for both the main coronary artery (main) and specific branch/segment (sub).

A ‘no culprit’ label was used when no culprit could be identified. Multiple labels were permitted only for the culprit-vessel category [e.g. right coronary artery (RCA)/ramus descendants anterior (RDA)]; all other categories were single-label. Transthoracic echocardiography reports were labelled for left ventricular (LV) systolic function and valvular dysfunction, including the type and severity for each cardiac valve [aortic (AV), mitral (MV), tricuspid (TV), and pulmonary (PV)], using guideline-consistent ordered categories: LV function was categorized as normal, mildly, moderately, or severely reduced, and valvular stenosis/regurgitation as none, mild, moderate, or severe.^12,13^ When multiple gradings were present within a report, the most severe category was selected. A ‘no data’ label was assigned when relevant findings were absent from the report. A complete overview of all label categories is provided in *Table 1*.

**Table 1:** Overview of the possible invasive coronary angiography and transthoracic echocardiography labels

Label	Values
ICA dataset	
Occlusion	Yes, no
No CAD	Yes, no
Graft	Yes, no
Treatment strategy	PCI, CABG, medical
Main	RDA, RCA, RCx, LM, Graft, IM, no
Sub	RDA, RCA, RCx, LM, Graft, IM, AL, D, MO, PL, RDP, RV, S, no
TTE dataset	
LV function	Normal, mildly, moderately, or severely reduced, no data
Valve dysfunction type	None, stenosis, regurgitation, both, no data
Valve dysfunction grade	None, mild, moderate, severe, no data

### Large language model assessment

**Figure 1:**
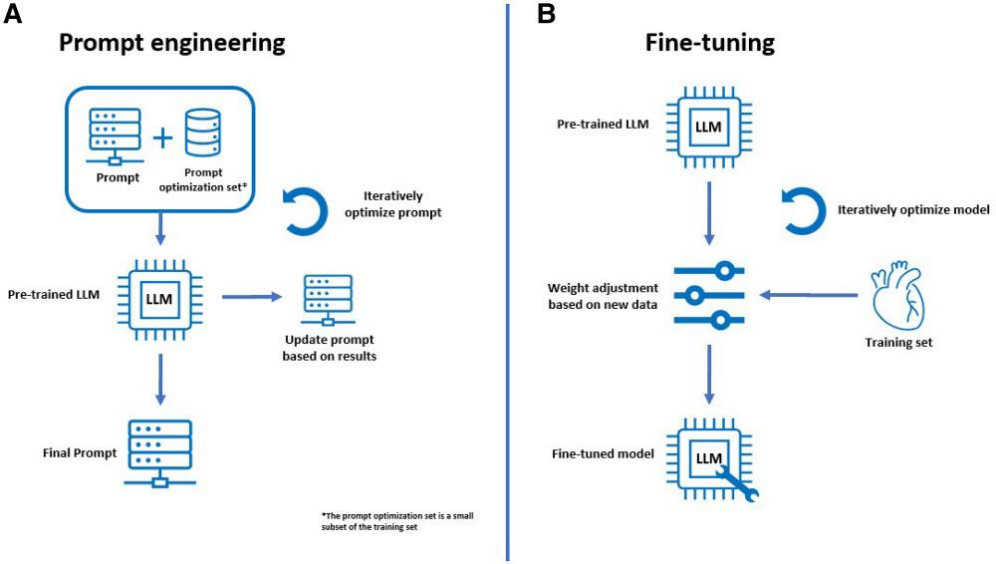
(*A*) Prompt engineering method, resulting in an optimized prompt, where no model training takes place. (*B*) Fine-tuning method, a pre-trained large language model is trained on a domain-specific dataset, where adjusting the weights of the network results in a fine-tuned model on labelled data to adjust to the target task.^14–16^ A brief schematic comparing prompt engineering and fine-tuning is provided in this image. Their classification performance and implementation complexity were systematically compared.

Both fine-tuning and prompt engineering (see *Figure 1*) were tested on a commercially available LLM (GPT-4o via Azure OpenAI) and several open-source state-of-the-art (SOTA) models available via Huggingface.co and Ollama.com that can be used on-site. Open-source SOTA models were selected by identifying the two most popular models for text classification or feature extraction across three categories: general-purpose, medical domain-specific, and multi-lingual LLMs. For on-site prompt engineering, two different hardware constraints are tested. A smaller local graphics processing unit (GPU) of 16 Gb, which would be an average laptop GPU and a bigger high-performance cluster (HPC) GPU of 48 Gb. For on-site fine-tuning only the HPC hardware was tested, since hardware requirements for fine-tuning are too large for small GPUs. Selection was done with the important constraint that the model could either be run (i) locally on a 16 Gb GPU for local prompt engineering or (ii) on a 48 Gb GPU for HPC prompt engineering or (iii) be trained on a 48 Gb GPU for fine-tuning.

#### Fine-tuning

For on-site fine-tuning, each model was extended with a classification layer, such that the model directly outputs class indexes. This classification layer was trained without pre-training, while the underlying model was fine-tuned on the manually labelled ICA and TTE datasets. To mitigate overfitting, we implemented early stopping based on the performance on the validation set, along with weight decay as a regularization technique. Additionally, the learning rate, a crucial hyperparameter, is fine-tuned to optimize performance while maintaining stability. For on-site fine-tuning, the following pre-trained models were selected from Huggingface.co on 2 March 2025: multi-lingual-e5-large from Intfloat^17^ (Multi-lingual 1), bert-base-multi-lingual-uncased-sentiment from NLPTown (Multi-lingual 2), BiomedVLPBioVil-T from Microsoft^18^ (Medical 1), MedCPT-Cross-Encoder from NCBI^19^ (Medical 2), bart-base from Facebook^20^ (General 1), and ms-marco-MiniLM-L-6-v2 from Cross-encoder (General 2).

Fine-tuning of the commercially available GPT-4o model was performed using the Azure OpenAI fine-tuning application programming interface (API). The model was adapted to the classification tasks using manually labelled reports. The fine-tuning process is subjected to pre-defined limitations inherent to the commercial API, which permits adjustment of only a single relevant hyperparameter; the learning rate multiplier. Modification of the model architecture or implementation of training optimizations such as early stopping or weight regularization is not possible. The optimal learning rate was selected based on validation performance and training stability, ensuring generalization to unseen data. Because fine-tuning was performed on OpenAI servers, the resulting model weights are not accessible to the authors and therefore cannot be shared.

#### Prompt engineering

Prompt-based inference was conducted using a few-shot prompting strategy. Each clinical report was combined with standardized labelling instructions to create a structured input format. Prompts were category-specific, with one call per label. Structured output templates (JSON schemas) were used to ensure consistency and facilitate accurate parsing. Prompts were iteratively refined on a subset of the training data, with adjustments to wording and structure aimed at minimizing misclassification and improving labelling accuracy. Model settings were configured to promote consistent outputs, with parameter settings to minimize variability during output generation. For local prompt engineering, the following small pre-trained models were selected from Ollama.com on 2 March 2025: Aya 23 from Cohere^21^ (Multi-lingual 1), Llama3.2 from Meta (Multi-lingual 2), MedLlama2 by Sourcell (Medical 1), Phi-4 from Microsoft (General 1), and Gemma3 from Google (General 2/multi-lingual). No second medical model was selected because a decent medical runner up model was not available at this moment. For HPC prompt engineering, the following larger pre-trained models were selected from Ollama.com on 2 March 2025: Gemma3 from Google (Multi-lingual 1) and Wizardlm2 from Microsoft (Multi-lingual 2), Meditron (Medical 1),^22^ Medllama2 by Sourcell (Medical 2), Deepseek-R1-Distill-Qwen from DeepSeek (General 1), and Phi-4 from Microsoft (General 2). For some models larger versions exist, the largest version that can fit on either the local GPU (16 Gb) or the HPC cluster GPU (48 Gb) was chosen.

### Analysis

Both data sets were randomly divided into a training and a test set, at patient level, with a ratio of 70:30. For the prompt engineering method, the training set was used for prompt optimization and example selection. For the fine-tuning method, the training set was again split, at patient level, in a training and validation set with a ratio of 85:15. The training set was used to fine-tune the models, while the validation set is used to monitor overfitting. For all on-site model comparisons, a five-fold cross-validation scheme was used. Model evaluation was done by calculating metrics on the combined predictions on all validation sets, which aggregate to the whole training set. The test set, which remains completely unseen during prompt optimization and fine-tuning, was reserved for final performance evaluation. Models were evaluated using the following performance metrics: accuracy, average recall, and macroaveraged F1-score. For the multi-class multi-label tasks (main and sub-culprit vessel), a strict evaluation criterion was adopted: classification outputs were only considered correct if the entire set of predicted labels per report exactly matched the reference labels. Consequently, partially correct classifications, such as predicting ‘RDA/D’ when only ‘D’ was correct, were seen as incorrect. To quantify variability, 95% confidence intervals (CIs) were estimated using 1000 bootstrap iterations for each metric. To assess statistically significant differences in performance, the Bonferroni corrected *P*-value (*P_b_*) is calculated per-label category, using the best-performing model as the reference for all pairwise comparisons. Finally, misclassified cases from the test set were manually reviewed to identify recurring patterns and potential sources of model error.

## Results

### Study population

Both datasets comprised 1000 unique reports. In the ICA dataset, each report represented a unique patient (*n* = 1000), while in the TTE dataset, the reports corresponded to 736 unique patients, with 264 patients contributing two reports each. No reports were duplicated and no patient appeared more than twice within the TTE dataset. The mean age of the patients was 65.7 ± 11.9 years in the ICA cohort and 62.2 ± 11.2 years in the TTE cohort. The sex distribution in the ICA dataset was 70.7% male (*n* = 707) and 29.3% female (*n* = 293). In the TTE dataset, 74.6% were male (*n* = 746) and 25.4% were female (*n* = 254). The initial clinical presentation among patients in the ICA dataset was ST-segment elevation myocardial infarction (STEMI) in 33.1% (*n* = 331), non-ST-segment elevation myocardial infarction (NSTEMI) in 30.7% (*n* = 307), and unstable angina in 36.2% (*n* = 362). In the TTE dataset, 66.2% (*n* = 487) of cases presented with STEMI, 25.7% (*n* = 189) with NSTEMI, and 8.2% (*n* = 60) with unstable angina.

### Data labelling

The ground truth label distributions across all variables in the ICA dataset were skewed, with varying degrees of class imbalance, ranging from 88 vs. 12% for the no CAD label to 33 vs. 67% for the occlusion label. As expected, the proportion of patients without CAD was low (12%), as most patients had an occlusion (67%), underwent PCI (75%), and did not have grafts (85%). In the culprit-vessel labels, the RDA,RCA, and ramus circumflexes (RCx) were the most frequently identified vessels in both the main (361, 211, and 318 times, respectively) and sub-segment categories (321, 291, and 147, respectively). In contrast, the anterolateral branch (AL) was not annotated in any case and other vessels such as the septal (S) and right ventricular (RV) branches were only rarely identified (both two times). A full overview of the label distributions is shown in the Supplementary material.

Label imbalance in the TTE dataset was even more pronounced. The most frequent class for LV function was ‘mildly impaired’ (52%) whereas ‘severely impaired’ LV function was observed in only 2% of cases. Complete data on LV function were available for all patients. Across all valves, ‘no dysfunction’ was the most common label (83%), followed by ‘regurgitation’ (overall 11%) in all but the PV. The PV had the highest proportion of missing data (9%). Notably, no cases of isolated stenosis were recorded for the MVs, TVs, or PVs. For valve dysfunction grading, the most common non-normal category was ‘mild’ (5%), while ‘moderate’ (*<*1%, 59 cases) and ‘severe’ (*<*1%, 12 cases) grades were uncommon and for mitral stenosis, pulmonary stenosis, and pulmonary regurgitation, and entirely absent for tricuspid stenosis. A full overview of the TTE label distributions is provided in the Supplementary material.

#### Annotator agreement

The average agreement between both annotators was high, as shown in *Tables 2* and *3*. In the ICA dataset, accuracy ranged from 0.917 to 0.987, average recall from 0.787 to 0.964 and F1 scores from 0.818 to 0.963. The highest scores were observed for graft presence, while the lowest were found in sub-segment-level vessel classification. In the TTE dataset, manual annotation scores were consistently high across all labels, with accuracy ranging from 0.993 to 1.00, average recall from 0.975 to 1.00 and F1 scores from 0.920 to 1.00. Human performance was particularly strong for binary labels in the ICA dataset and for all TTE labels, while greater variability was observed in more complex and ambiguous tasks such as culprit-vessel annotation.

**Table 2:** Model comparison for invasive coronary angiography report classification on the test set

Label	Accuracy	Avg. recall	F1
**Main** *(manual)*	*0.943*	*0.920*	*0.963*
FT (Intfloat)	0.634 (0.580–0.683)	0.392 (0.362–0.42)	0.718 (0.672–0.764)
FT (GPT-4o)	**0.870 (0.830–0.907)**	**0.830 (0.774–0.888)**	**0.937 (0.914–0.958)**
PE (Phi-4)	0.522 (0.467–0.580)	0.510 (0.449–0.579)	0.715 (0.671–0.757)
PE (GPT-4o)	0.761 (0.710–0.803)	0.768 (0.693–0.838)	0.881 (0.849–0.91)
**Sub** *(manual)*	*0.917*	*0.787*	*0.941*
FT (Intfloat)	0.533 (0.477–0.587)	0.212 (0.185–0.252)	0.607 (0.554–0.658)
FT (GPT-4o)	**0.746 (0.693–0.793)**	0.668 (0.576–0.764)	**0.819 (0.776–0.857)**
PE (Phi-4)	0.460 (0.403–0.520)	0.611 (0.528–0.697)	0.797 (0.761–0.833)
PE (GPT-4o)	0.634 (0.580–0.687)	0.795* (0.719–0.872)	**0.830 (0.799–0.862)**
**No CAD** *(manual)*	*0.974*	*0.964*	*0.945*
FT (Intfloat)	0.960* (0.937–0.980)	0.883 (0.820–0.939)	0.918* (0.867–0.959)
FT (GPT-4o)	0.964* (0.940–0.983)	0.969* (0.940–0.990)	0.936* (0.897–0.971)
PE (Phi-4)	0.974* (0.953–0.990)	0.899 (0.865–0.928)	0.782 (0.727–0.838)
PE (GPT-4o)	0.973* (0.953–0.990)	0.950* (0.904–0.985)	0.949* (0.913–0.979)
**Occlusion** *(manual)*	*0.926*	*0.933*	*0.917*
FT (Intfloat)	0.793 (0.750–0.837)	0.752 (0.701–0.801)	0.761 (0.710–0.810)
FT (GPT-4o)	0.920* (0.89–0.947)	0.920* (0.887–0.951)	0.912* (0.878–0.943)
PE (Phi-4)	0.761 (0.713–0.81)	0.697 (0.646–0.752)	0.709 (0.654–0.767)
PE (GPT-4o)	0.927* (0.897–0.953)	0.917* (0.881–0.949)	0.919* (0.884–0.949)
**Treatment strategy** *(manual)*	*0.976*	*0.924*	*0.818*
FT (Intfloat)	0.953* (0.930–0.977)	0.895* (0.825–0.955)	0.882* (0.810–0.940)
FT (GPT-4o)	0.987* (0.973–0.997)	0.970* (0.926–0.998)	0.97* (0.931–0.997)
PE (Phi-4)	0.940 (0.910–0.967)	0.835 (0.748–0.917)	0.847* (0.762–0.919)
PE (GPT-4o)	0.977* (0.957–0.993)	0.915* (0.841–0.976)	0.930* (0.868–0.977)
**Graft** *(manual)*	*0.987*	*0.940*	*0.957*
FT (Intfloat)	0.970* (0.950–0.987)	0.901* (0.839–0.959)	0.930* (0.881–0.970)
FT (GPT-4o)	0.996* (0.990–1.00)	0.998* (0.994–1.00)	0.992* (0.975–1.00)
PE (Phi-4)	0.993* (0.983–1.00)	0.924* (0.864–0.974)	0.947* (0.905–0.981)
PE (GPT-4o)	0.993* (0.983–1.00)	0.996* (0.990–1.00)	0.986* (0.965–1.00)

Four LLM models and training methods are compared on six labels extracted from ICA reports. Comparison is done with three metrics: accuracy, average recall, and F1. The 95% CI is given in brackets. The best metric score and any score that is not significantly different from this score (*P_b_* = 0.0167), is indicated with bold font. Manual annotation scores are averaged human annotator scores and are shown in *italic*. Significant improvement or equality to the manual score is indicated with an asterisk (*).

FT, fine-tuning; PE, prompt engineering.

**Table 3:** Model comparison for transthoracic echocardiography report classification on the test set

Label	Accuracy	Avg. recall	F1
**LV function** *(manual)*	*0.997*	*0.998*	*0.998*
FT (Intfloat)	0.984* (0.967–0.997)	**0.918 (0.809–0.988)**	**0.947 (0.869–0.993)**
FT (GPT-4o)	1.00* (1.00–1.00)	1.00* (1.00–1.00)	1.00* (1.00–1.00)
PE (Phi-4)	0.727 (0.667–0.777)	0.805 (0.753–0.849)	0.518 (0.429–0.647)
PE (GPT-4o)	0.997* (0.990–1.00)	0.998* (0.995–1.00)	0.993* (0.976–1.00)
**Mitral Sten. Grade** *(manual)*	*1.00*	*1.00*	*1.00*
FT (Intfloat)	0.969 (0.950–0.987)	0.555 (0.495–0.667)	0.578 (0.488–0.745)
FT (GPT-4o)	0.997* (0.990–1.00)	0.998* (0.995–1.00)	0.972* (0.897–1.00)
PE (Phi-4)	0.796 (0.750–0.837)	0.410 (0.388–0.431)	0.223 (0.215–0.229)
PE (GPT-4o)	1.00* (1.00–1.00)	1.00* (1.00–1.00)	1.00* (1.00–1.00)
**Mitral Reg. grade** *(manual)*	*0.997*	*0.975*	*0.920*
FT (Intfloat)	0.866 (0.827–0.903)	0.372 (0.298–0.475)	0.379 (0.295–0.504)
FT (GPT-4o)	0.990* (0.977–1.00)	0.963* (0.900–1.00)	0.896* (0.738–1.00)
PE (Phi-4)	0.893 (0.860–0.927)	0.783 (0.688–0.869)	0.707 (0.532–0.853)
PE (GPT-4o)	**0.980 (0.963–0.993)**	0.946* (0.878–0.989)	0.897* (0.743–0.993)
**Aortic Sten. grade** *(manual)*	*1.00*	*1.00*	*1.00*
FT (Intfloat)	0.907 (0.873–0.940)	0.219 (0.200–0.250)	0.208 (0.187–0.242)
FT (GPT-4o)	0.997* (0.987–1.00)	0.999* (0.997–1.00)	0.991* (0.968–1.00)
PE (Phi-4)	0.866 (0.823–0.903)	0.791 (0.721–0.850)	0.516 (0.377–0.665)
PE (GPT-4o)	0.990* (0.977–1.00)	0.856* (0.753–1.00)	0.836* (0.712–1.00)
**Aortic Reg. grade** *(manual)*	*0.993*	*0.984*	*0.991*
FT (Intfloat)	0.843 (0.800–0.883)	0.201 (0.200–0.201)	0.184 (0.178–0.19)
FT (GPT-4o)	0.987* (0.973–0.997)	0.975* (0.948–0.999)	0.978* (0.952–0.996)
PE (Phi-4)	0.876 (0.837–0.913)	0.795 (0.772–0.834)	0.661 (0.553–0.744)
PE (GPT-4o)	**0.973 (0.953–0.990)**	**0.885 (0.751–0.967)**	**0.904 (0.742–0.980)**
**Tricuspid Sten. grade** *(manual)*	*0.993*	*0.997*	*0.957*
FT (Intfloat)	0.963 (0.94–0.983)	0.500 (0.500–0.500)	0.491 (0.485–0.496)
FT (GPT-4o)	0.967 (0.947–0.987)	0.983 (0.972–0.993)	0.831 (0.727–0.925)
PE (Phi-4)	0.838 (0.793–0.877)	0.435 (0.415–0.454)	0.185 (0.177–0.229)
PE (GPT-4o)	0.997* (0.990–1.00)	0.998* (0.995–1.00)	0.977* (0.913–1.00)
**Tricuspid Reg.grade** *(manual)*	*0.993*	*0.998*	*0.978*
FT (Intfloat)	0.887 (0.850–0.923)	0.260 (0.250–0.333)	0.245 (0.230–0.317)
FT (GPT-4o)	**0.963 (0.943–0.983)**	**0.978 (0.947–0.994)**	**0.905 (0.841–0.955)**
PE (Phi-4)	0.853 (0.81–0.893)	0.797 (0.702–0.878)	0.491 (0.360–0.657)
PE (GPT-4o)	0.980* (0.963–0.993)	**0.961 (0.912–0.997)**	0.901* (0.701–0.985)
**Pulmonary Sten. grade** *(manual)*	*0.997*	*0.998*	*0.991*
FT (Intfloat)	0.987* (0.973–0.997)	0.950* (0.896–0.998)	0.963* (0.926–0.992)
FT (GPT-4o)	0.993* (0.983–1.00)	0.982* (0.945–1.00)	0.982* (0.950–1.00)
PE (Phi-4)	0.834 (0.790–0.877)	0.508 (0.459–0.568)	0.260 (0.191–0.384)
PE (GPT-4o)	0.996* (0.987–1.00)	0.998* (0.993–1.00)	0.990* (0.967–1.00)
**Pulmonary Reg. grade** *(manual)*	*0.997*	*0.999*	*0.994*
FT (Intfloat)	0.963 (0.940–0.983)	0.634 (0.597–0.665)	0.638 (0.612–0.659)
FT (GPT-4o)	0.990* (0.977–1.0)	0.939* (0.820–1.00)	0.958* (0.873–1.00)
PE (Phi-4)	0.816 (0.770–0.860)	0.740 (0.684–0.800)	0.427 (0.349–0.507)
PE (GPT-4o)	0.993* (0.983–1.00)	0.950* (0.831–1.00)	0.964* (0.876–1.00)

Four LLM models and training methods are compared on nine labels extracted from TTE reports. Comparison is done with three metrics: accuracy, average recall, and F1. The 95% CI is given in brackets. The best metric score and any score that is not significantly different from this score (*P_b_* = 0.0167), is indicated with bold font. Manual annotation scores are averaged human observer scores and are shown in *italic*. Significant improvement or equality to this manual score is indicated.

### Prompt engineering model assessment

**Figure 2:**
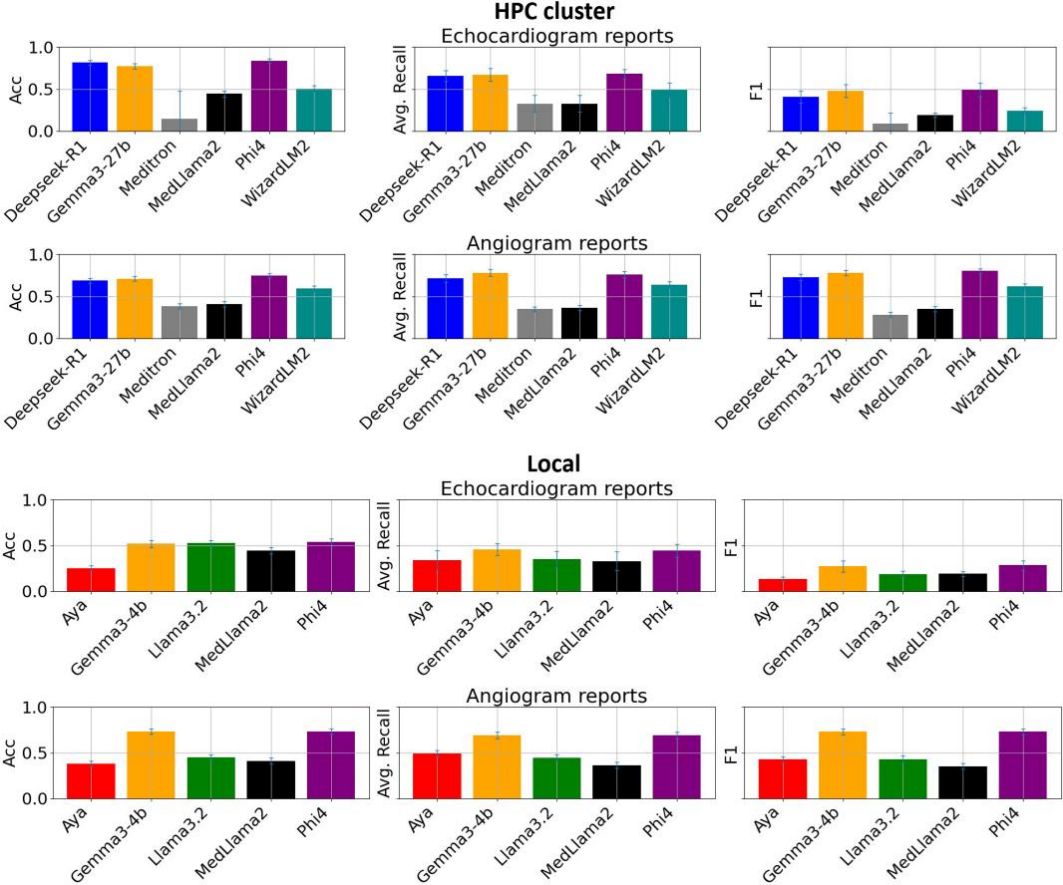
Model comparisons for prompt engineering on a local machine (16 Gb) and on a high-performance cluster cluster (48 Gb) for invasive coronary angiography and transthoracic echocardiography report classification. For each model the average performance (of all labels) is shown. 95% confidence interval of metrics calculated with 1000 bootstrapped samples is indicated with error bars.

All inputs, combination of prompt and report, were below the context limit and no truncation was encountered. *Figure 2* shows the comparison of the six selected LLMs for the classification of ICA and TTE reports. The metrics reflect the average classification performance across all labels per report. Results represent the aggregated outcomes from five-fold cross-validation conducted on the training/validation dataset. Original values are five-fold cross-validation means on training/validation set, see *Figures 5–10* in the Supplementary material. For each metric, the mean value and the corresponding 95% CI, derived from 1000 bootstrap samples, are shown. Models run on a local smaller GPU demonstrated slightly inferior performance compared with those trained on a HPC cluster, although the difference was modest. Notably, both the Gemma3 and the Phi4 models showed robust performance in both GPU environments, with the Phi4 model run on the HPC cluster showing best overall performance.

### Fine-tune model assessment

**Figure 3:**
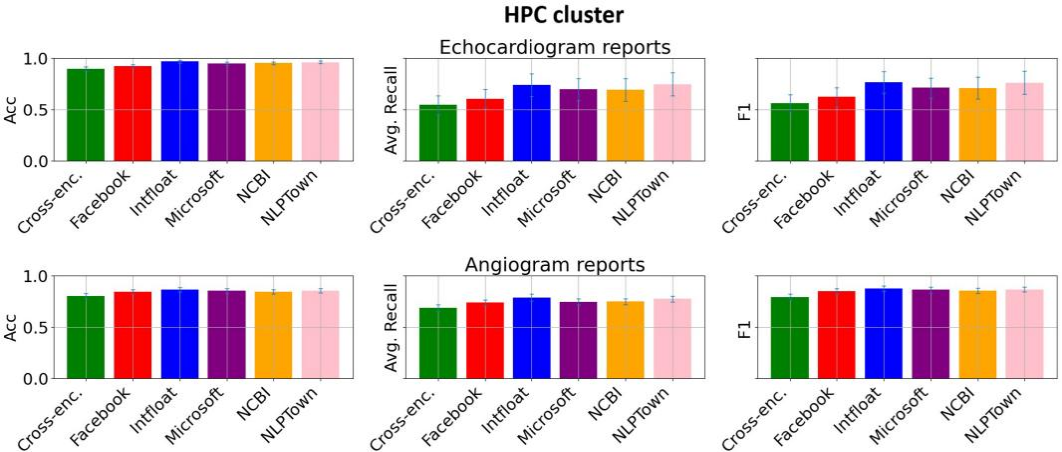
Model comparison for fine-tuning on a high-performance cluster cluster on invasive coronary angiography and transthoracic echocardiography reports. For each model, the average performance (of all labels) is shown. 95% confidence interval of metrics calculated with 1000 bootstrapped samples is indicated with error bars.

For on-site fine-tuning models, context limit was reached in 2% of cases, with no difference in performance for various truncation methods. For the commercial fine-tuning model no truncation was encountered. *Figure 3* shows the comparison of the six selected LLMs for the classification of ICA and TTE reports. For each model the average performance (of all labels) is shown. 95% CI of metrics calculated with 1000 bootstrapped samples is indicated with error bars. The multi-lingual model from Intfloat showed best overall performance for classification of both report types.

### Classification results and model comparison

*Tables 2* and *3* show the performance of the best local prompt engineering and fine-tuning models compared with prompt engineering and fine-tuning with the commercial model (GPT-4o) on the held-out test set. The average human annotator performance per task is shown as an indicator of task difficulty. Per-label counts for the whole dataset are provided in Supplementary material online, *Figures S1*–*S4*, which contextualize the class imbalance underlying *Tables 2* and *3*. Especially, for the more difficult tasks, GPT-4o showed superior performance for both prompt engineering and fine-tuning methods and often approaches or surpasses the human annotator benchmark. For most labels, no statistically significant difference was observed between prompt engineering and fine-tuning when applied to GPT-4o. In contrast, performance differences were more variable across tasks when using the open-source SOTA models, while prompt engineering and fine-tuning yielded comparable average performance, outcomes varied depending on the task. Prompt engineering tended to underperform when the test set lacked representation of categories included in the prompt. Fine-tuning on the other hand exhibited diminished performance in severely imbalanced datasets. Complex tasks such as culprit-vessel detection, which are multi-class and multi-label tasks, are challenging for all on-site models, regardless of training strategy.

### Misclassification analysis

All misclassified labels in the held-out test set of 300 ICA and 300 TTE reports were manually reviewed to identify recurring patterns and potential sources of model error.

#### Open-source models

For the ICA report classification (*Table 2*), the fine-tuned model most frequently produced false negatives for ‘no CAD’ (11/300, 4%), while prompt engineering yielded a higher false-positive rate (46/300, 15%). Both models over predicted occlusions (13–18%). Graft detection was generally reliable. However, the fine-tuned model systematically missed isolated venous grafts (*n* = 9/300, 3%), while prompt-engineering errors were more evenly distributed. For ‘Treatment strategy’ classification, the fine-tuned model tended to over predict referral for CABG (*n* = 5/300, 2%), whereas the prompt-engineered model more often missed true PCI cases (*n* = 8/300, 3%). Culprit-vessel classification showed notable limitations. The fine-tuned model only correctly identified 2/24 multi-vessel culprit cases, and this pattern was not observed with prompt engineering, though its overall accuracy was inferior in this category. Among main culprit vessel, 16 (5%) errors of fine-tuned model and 26 (9%) errors with prompt engineering were associated to graft-related misclassification. Notably, prompt engineering failed to assign the ‘no culprit’ label in any case, despite its presence in 52 test samples. In the sub-vessel category, the fine-tuned model only assigned the labels RCA, RCx, RDA, and no.

In TTE reports (*Table 3*), the fine-tuned model most frequently misclassified mild re-with an gurgitation as none (AV: *n* = 25/300, 8%; MV: *n* = 14/300, 5%). In contrast, prompt engineering occasionally overestimated pathology, predicting dysfunction where none was present. Stenosis grading errors followed similar patterns, with the fine-tuned model under-calling and prompt-engineering over-calling severity. Notably, the fine-tuned model assigned the ‘no dysfunction’ label to the AV in all cases.

#### Commercial models

For commercial models (*Table 2*), both prompt engineering and fine-tuning showed the lowest performance for culprit-vessel classification. Prompt engineering produced 24% and the fine-tuned model 13% main vessel labelling errors. Common issues included labelling both main and sub-branches (15 and 12%, respectively), and misclassifying graft vs. native vessels. Both models consistently failed to recognize the IM branch correctly (3/9 cases each). Errors in classifying the presence of acute occlusions were often related to the presence of chronic total occlusion (CTO)s and grafts. Misclassifications also occurred in differentiating pre-existing CTOs from new obstructive disease. In both approaches, the most common misclassifications for the labels graft, no CAD, and occlusion were false positives. With the prompt engineering method, these occurred in 2/300 (1%), 6/300 (2%), and 19/300 (6%) cases, respectively, whereas with the fine-tuned model, the corresponding rates were 1/300 (*<*1%), 10/300 (3%), and 6/300 (2%). For ‘treatment strategy’ classification, the most common error in both models was predicting medical therapy only while a referral for CABG was mentioned, particularly in reports indicating pending diagnostics (prompt engineering: *n* = 5/300, 2%; fine-tuned model: *n* = 2/300, 1%).

In TTE data (*Table 3*), valvular assessment was the main challenge, particularly differentiating between ‘none’ and ‘no data’ due to inconsistencies between structured data and narrative conclusions. For example, cases where mild mitral regurgitation was quantitatively reported but summarized as ‘no dysfunction’ in the narrative conclusion. Additional errors were linked to ambiguous or non-diagnostic phrases, such as ‘TV: TR gradient 30 mmHg’ or ‘AV opens well visually, no reliable gradient’. Both models under predicted mild regurgitation, with the fine-tuned model showing fewer errors overall. Left ventricular function classification was accurate, with only isolated errors.

## Discussion

Large language model-based methods are a very valuable tool in the automation of ICA and TTE report classification. Depending on the accuracy required, the labelling difficulty and the available budget and computational power, different LLM-based methods can be used for the automation of report classification.

In this study, the performance of LLMs using both prompt engineering and fine-tuning was evaluated for the classification of TTE and ICA reports across different computational environments: (i) a local server, (ii) a HPC cluster, and (iii) a commercial cloud-based API (e.g. GPT-4o). The goal was to explore the practical feasibility, performance, and trade-offs for different LLM approaches in processing real-world cardiology data.

### Model performance and comparative analysis

Across platforms, LLMs demonstrated robust performance in classifying key clinical findings with minimal pre-processing, underscoring their potential integration into cardiology workflows such as automated EHR annotation, registry data generation, and retrospective data structuring. Compared with manual annotation by a single experienced reviewer, GPT-4o-based approaches demonstrated similar performance for most tasks, except for culprit-vessel identification, the most complex task, and tricuspid regurgitation grading with the fine-tuning approach, where severe class imbalance impaired model performance. Open-source models exhibited similar task-specific performance trends but with consistently lower accuracy. For simpler tasks with balanced classes, both modelling approaches performed comparable to the human annotator. The lowest performance was observed in tasks involving both main and sub-culprit-vessel identification, consistent with the inherent complexity of the task. Additionally, the strict evaluation criterion for these tasks contributes to lower performance metrics. In the context of TTE reports, classification errors often resulted from inconsistencies between structured quantitative measurements and the narrative conclusions. For example, cases describing mild mitral regurgitation in the measurements section were occasionally summarized in the conclusion as ‘no dysfunction’. Furthermore, the presence of clinically benign phrases, such as ‘calcified annulus with normal function’, occasionally triggered false-positive classifications. The various approaches revealed different levels of task comprehension, likely influenced by model architecture and scale.^23^ For instance, the prompt-engineered open-source model frequently labelled any vessel with atherosclerotic plaque as a culprit lesion, without adequately considering the degree of luminal narrowing, indicating limitations in nuanced clinical reasoning. Importantly, GPT-4o achieved high and consistent performance across both prompt-based and fine-tuned implementations. However, fine-tuning required significantly greater computational resources, a larger volume of annotated training data and incurred substantially higher monetary costs. These trade-offs suggest that for many clinical applications, particularly in resource-constrained environments, prompt engineering with commercial models offers a more pragmatic and scalable solution. Conversely, in complex tasks such as culprit-vessel labelling, fine-tuning appeared to meaningfully enhance performance, supporting its use where feasible. While locally deployed open-source models did not match GPT-4o’s performance in complex classification tasks, they remain valuable in settings with simpler classification needs, stricter data privacy constraints, or limited budgets. These models provide a viable alternative for institutions that prefer not to rely on third-party cloud-based services.

### Prompt engineering vs. fine-tuning

A notable observation in our analysis was that smaller fine-tuned models achieved performance comparable to larger prompt-engineered models on several classification tasks. This aligns with findings from prior computational research, which suggest that, while fine-tuning allows for task-specific adaptation, its benefits may be constrained by the underlying model capacity.^24^ Conversely, larger prompt-engineered models can leverage broad pre-trained knowledge and perform well without full retraining. These results support a balanced interpretation: a more targeted learning strategy (fine-tuning) applied to a smaller model may yield comparable results to a less customized strategy (prompt engineering) implemented with a larger model. Additionally, it was shown that fine-tuning is more susceptible to class imbalance, which can lead to overfitting if not properly mitigated. This was particularly evident in the fine-tuned open-source model, which learned to classify all AVs as non-dysfunctional; a result that maximized performance on the imbalanced training set but failed to generalize. However, this holds true for all use cases with highly imbalanced data. In comparison, prompt-engineered approaches demonstrated greater resilience to label sparsity, suggesting an advantage in scenarios with limited or unevenly distributed annotations. From a practical perspective, this trade-off highlights the importance of aligning methodological choices with both task complexity and available infrastructure. While fine-tuning offers the potential for precise task optimization, it demands considerable computational resources, annotated training data, and technical expertise. In contrast, prompt engineering, particularly when applied to large-scale foundation models, offers a more accessible and scalable alternative, especially in structured domains such as diagnostic and procedural cardiology reporting.

The dataset used for training and evaluation reflects a typical ACS population, representative both in patient characteristics and disease distribution.^25^ This real-world alignment enhances the external validity of our findings. However, as a consequence, the dataset exhibits substantial class imbalance, mirroring the natural prevalence of clinical findings in ACS, which poses challenges for model training, particularly for fine-tuning strategies. While prompt-based approaches can partially mitigate these effects through tailored task formulations, fine-tuned models remain more susceptible to underperformance in underrepresented classes.

### Limitations and generalizability

Furthermore, overall human annotator scores were high, but lower for more complex labels such as culprit vessel and occlusion, likely due to the ambiguous and complex nature of these annotations. During ICA procedures, the culprit lesion may not be unequivocally identified, particularly in cases of NSTEMI, multi-vessel disease, or diffuse atherosclerosis.^26^ Labelling sub-branches of the culprit vessel was even more ambiguous, due to complex anatomical relations and lesions affecting multiple branches at the same time. These ambiguities, exacerbated by the multi-class and multi-label nature of vessel-related classifications, make for highly complex classification tasks. Additionally, a notable proportion of the dataset included patients with prior CABG or CTOs, introducing further anatomical variation and interpretation challenges.

To isolate model performance from labelling effort, we fixed the labelled dataset size across models and did not rebalance the dataset, which keeps implementation burden comparable but leaves some label categories with limited representation. In applied settings, rare labels may be enriched via keyword screening to prioritize these cases.

This study primarily focused on moderately structured clinical text, due to the procedural nature of analysed reports, which typically contain more templated or semi-standardized phrasing. Extending these methods to unstructured clinical narratives, such as clinical rounding notes or discharge summaries, will likely pose greater challenges due to their variability and contextual complexity. Nonetheless, reports used in this analysis were created by numerous different cardiologists, introducing heterogeneity in structure and phrasing.

We anticipate that similar LLM-based strategies can effectively be extended to less structured clinical text, provided that more extensive prompt engineering and carefully defined output schemas are employed to handle the greater variability. Moreover, while our datasets were sourced from a single academic institution, the underlying LLM-based approaches are inherently portable. They can be adapted to local data environments, allowing for institution-specific customization while leveraging generalizable architectures and workflows. Nevertheless, our comparisons were limited to single-centre datasets; extending these methods across multiple institutions may alter performance, as the benefits of fine-tuning could be reduced. Additionally, because we did not formally map outputs to standardized terminologies such as SNOMED CT, cross-institution interoperability remains to be established.

### Future directions

Future research should prioritize the development of advanced prompt optimization strategies, including automated prompt generation and hybrid frameworks that integrate prompt engineering with minimal fine-tuning. These approaches may offer a more efficient balance between performance and resource demands, particularly in settings with limited annotated data. In addition, performance could be further enhanced by incorporating post-processing techniques, such as rule-based corrections or ensemble methods that aggregate outputs from multiple models to enhance reliability. Guideline-grounded prompting and retrieval-augmented generation (RAG) may be explored to surface the most relevant guideline passages at inference time. When combined with schema-constrained outputs and simple rule-based adjudication, guideline-based diagnoses could be derived from free-text reports while maintaining transparency and ease of update.

The application of LLM-based data extraction to less structured clinical texts, such as progress notes and discharge summaries, warrants further investigation, as these sources contain rich contextual information but pose greater linguistic and structural challenges. Another interesting direction is applying LLMs to map free-text reports directly to standardized terminologies to streamline multi-institution data aggregation and enable joint analyses with minimal additional conversion steps.

Given the rapid evolution of LLM architectures and hardware, with new models showing both similar performance with a lower parameter count and superior performance with similar or higher parameter counts, we anticipate continuous improvement in performance, accessibility, and applicability in clinical cardiology settings.^27^

## Conclusions

Both fine-tuning and prompt engineering approaches to LLMs offer valuable tools for the structured classification of cardiology reports. Prompt engineering provides a lightweight, adaptable and cost-efficient strategy, particularly suitable for low-resource settings. Fine-tuning, while resource-intensive, enables more targeted optimization. While commercial LLMs generally outperform open-source models in challenging classification tasks, locally deployed models achieved good performance for more structured or narrowly defined applications. The choice between approaches should be guided by the clinical use case, available infrastructure, local expertise, and regulatory or privacy considerations. Critically, the traditional bottleneck of manual data labelling is rapidly becoming obsolete. With LLMs now capable of accurate, scalable information extraction from raw clinical text, the development of machine learning pipelines no longer hinges on large annotated datasets. This shift unlocks new opportunities for rapid deployment of AI in cardiology, from real-time decision support to large-scale data curation. As the technology continues to evolve, LLMs are set to become foundational infrastructure for cardiovascular research and clinical practice alike.

## Supplementary Material

ztaf127_Supplementary_Data

## Data Availability

The data underlying this article cannot be shared publicly due to the privacy of participants in the study.
