# Supplementary material for: Large language models for structured cardiovascular data extraction: a foundation for scalable research and clinical applications
Source: Eur Heart J Digit Health. 2025 Nov 14;7(2):ztaf127. doi: 10.1093/ehjdh/ztaf127 (PMC12893214; doi:10.1093/ehjdh/ztaf127)
Supplement: ztaf127_Supplementary_Data [file ztaf127_supplementary_data.zip › Appendix figures.docx]

Appendix figures:

Figure 4: Distribution of the manually assigned single-label labels for the ICA dataset.

Figure 5: Distribution of the manually assigned vessel labels.

Figure 6: Distribution of the manually assigned categorical LV function labels.

Figure 7: Distribution of the manually assigned categorical valve dysfunction labels.

Figure 8: Model comparison for the finetuning method on the TTE reports. Mean and 95% CI of 1000 bootstrap samples are shown. Results are aggregated metrics of 5 fold cross-validation on the training set.

Figure 9: Model comparison for the finetuning method on the ICA reports. Mean and 95% CI of 1000 bootstrap samples are shown. Results are aggregated metrics of 5 fold cross-validation on the training set.

Figure 10: Model comparison for local prompt engineering on TTE reports. Metrics show

aggregated values of 5-fold cross-validation on trainings set. 95% CI of metrics calculated with 1000 bootstrapped samples is indicated with error bars. Sten. = stenosis, reg. =

Regurgitation.

Figure 11: Model comparison for local prompt engineering on ICA reports. Metrics show

aggregated values of 5-fold cross-validation on trainings set. 95% CI of metrics calculated with 1000 bootstrapped samples is indicated with error bars.

Figure 12: Model comparison for HPC prompt engineering on TTE reports. Metrics show

aggregated values of 5-fold cross-validation on trainings set. 95% CI of metrics calculated with 1000 bootstrapped samples is indicated with error bars. Sten. = stenosis, reg. =

Regurgitation

Figure 13: Model comparison for HPC prompt engineering on ICA reports. Metrics show

aggregated values of 5-fold cross-validation on trainings set. 95% CI of metrics calculated with 1000 bootstrapped samples is indicated with error bars
