# Supplementary figures and images for: Large language models for structured cardiovascular data extraction: a foundation for scalable research and clinical applications
Source: Eur Heart J Digit Health. 2025 Nov 14;7(2):ztaf127. doi: 10.1093/ehjdh/ztaf127 (PMC12893214; doi:10.1093/ehjdh/ztaf127)

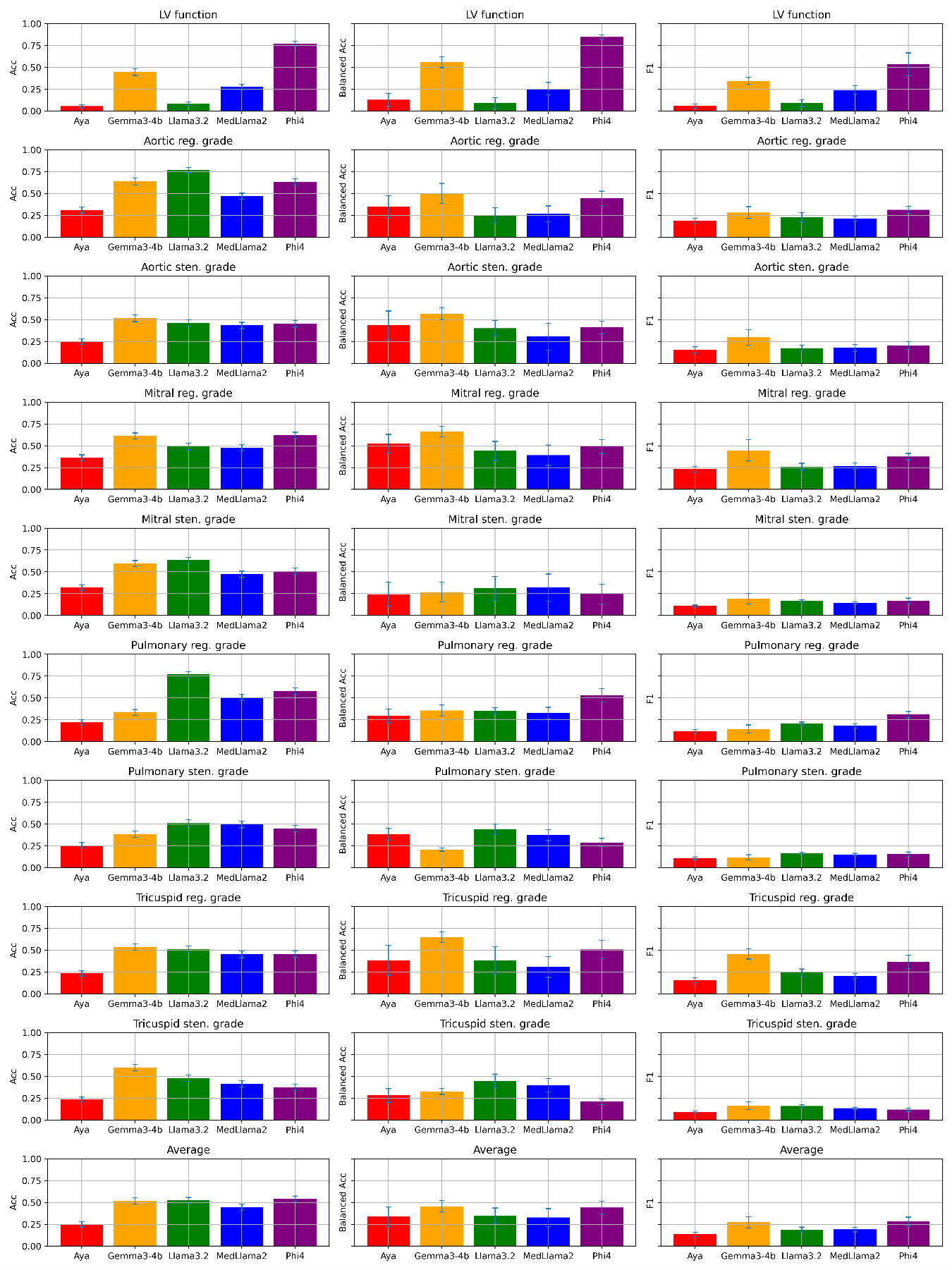

Supplement: ztaf127_Supplementary_Data [file ztaf127_supplementary_data.zip › SubFig7_LLM.png]

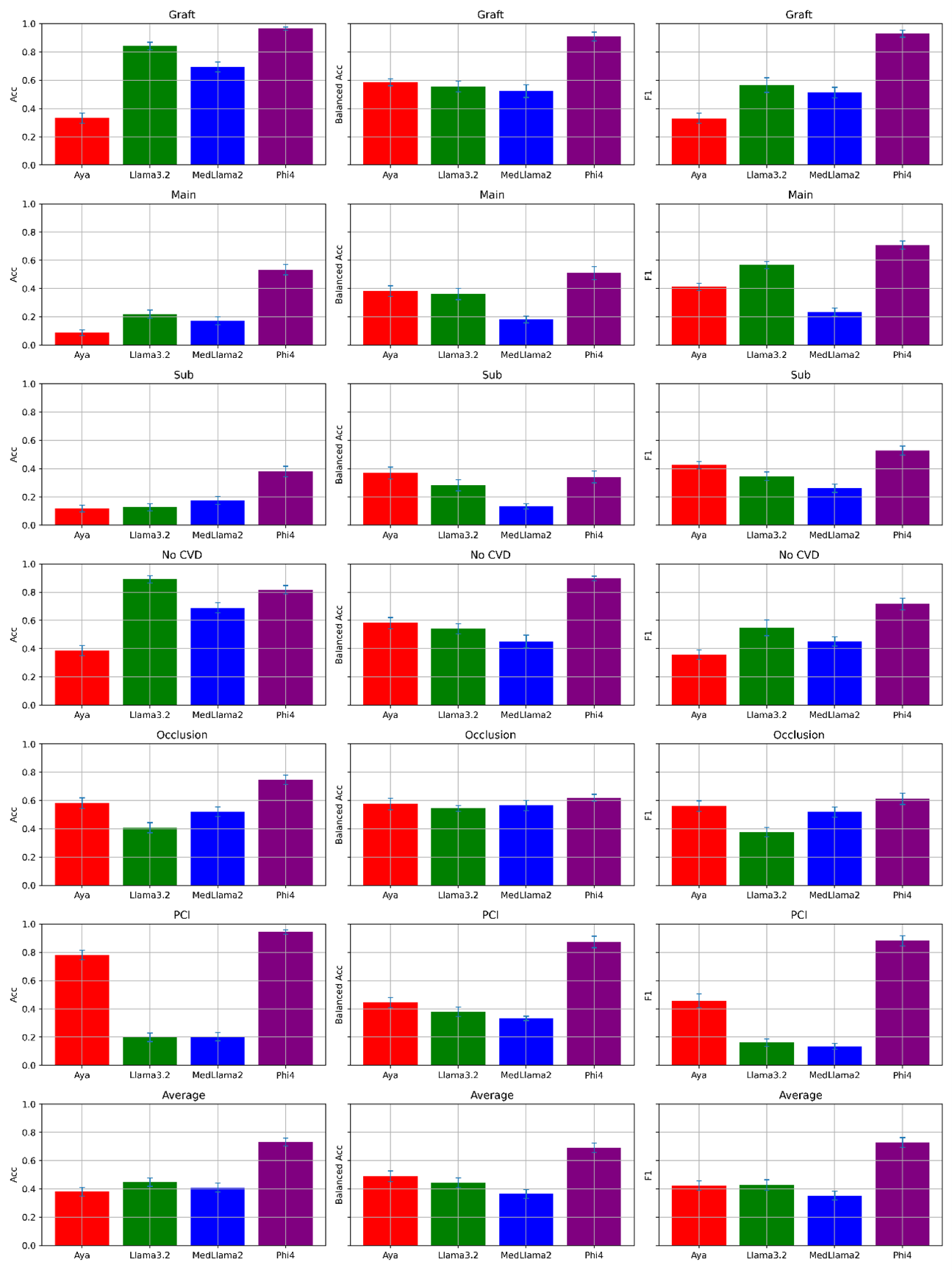

Supplement: ztaf127_Supplementary_Data [file ztaf127_supplementary_data.zip › SubFig8_LLM.png]

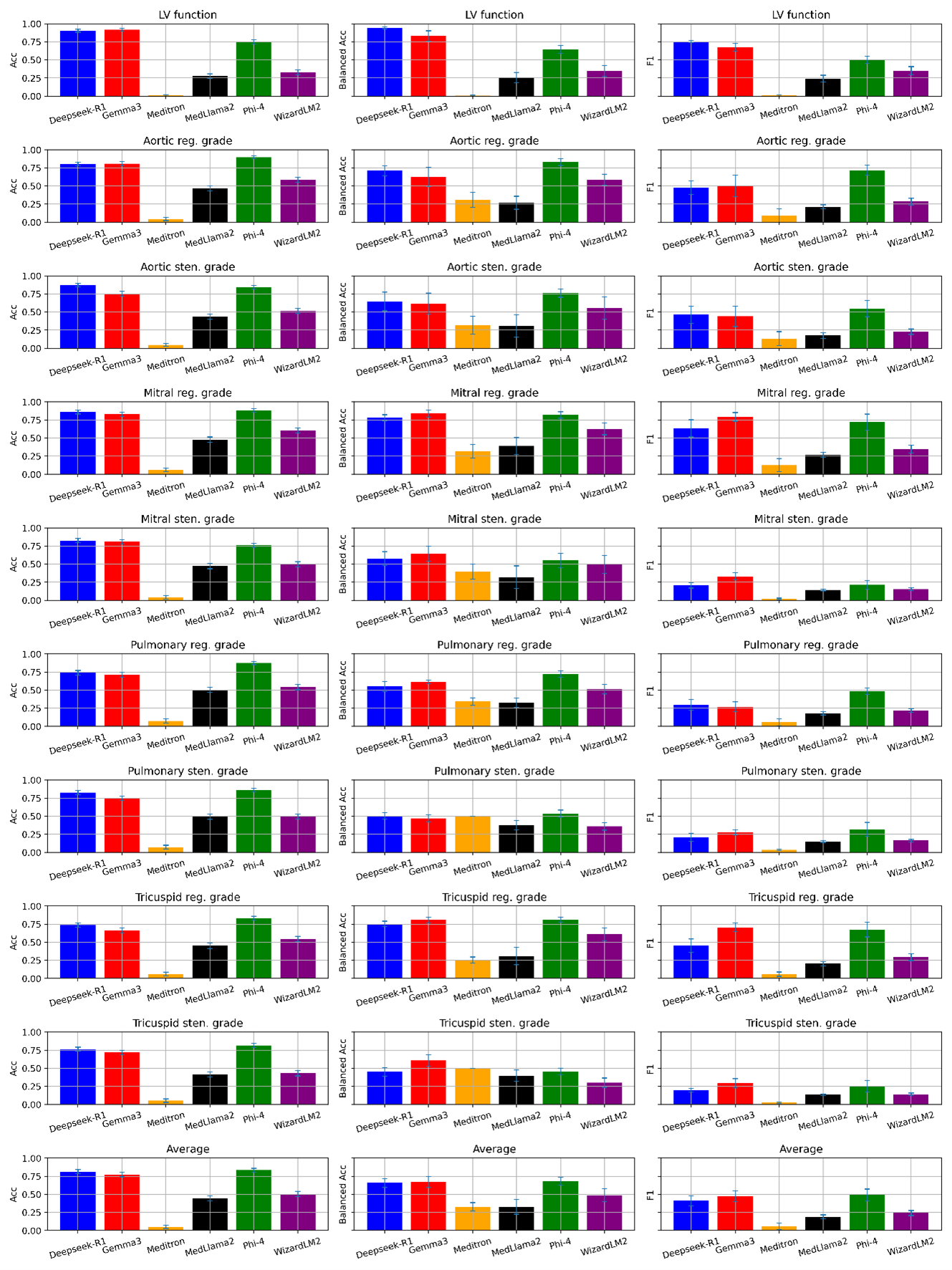

Supplement: ztaf127_Supplementary_Data [file ztaf127_supplementary_data.zip › SubFig9_LLM.jpg.png]

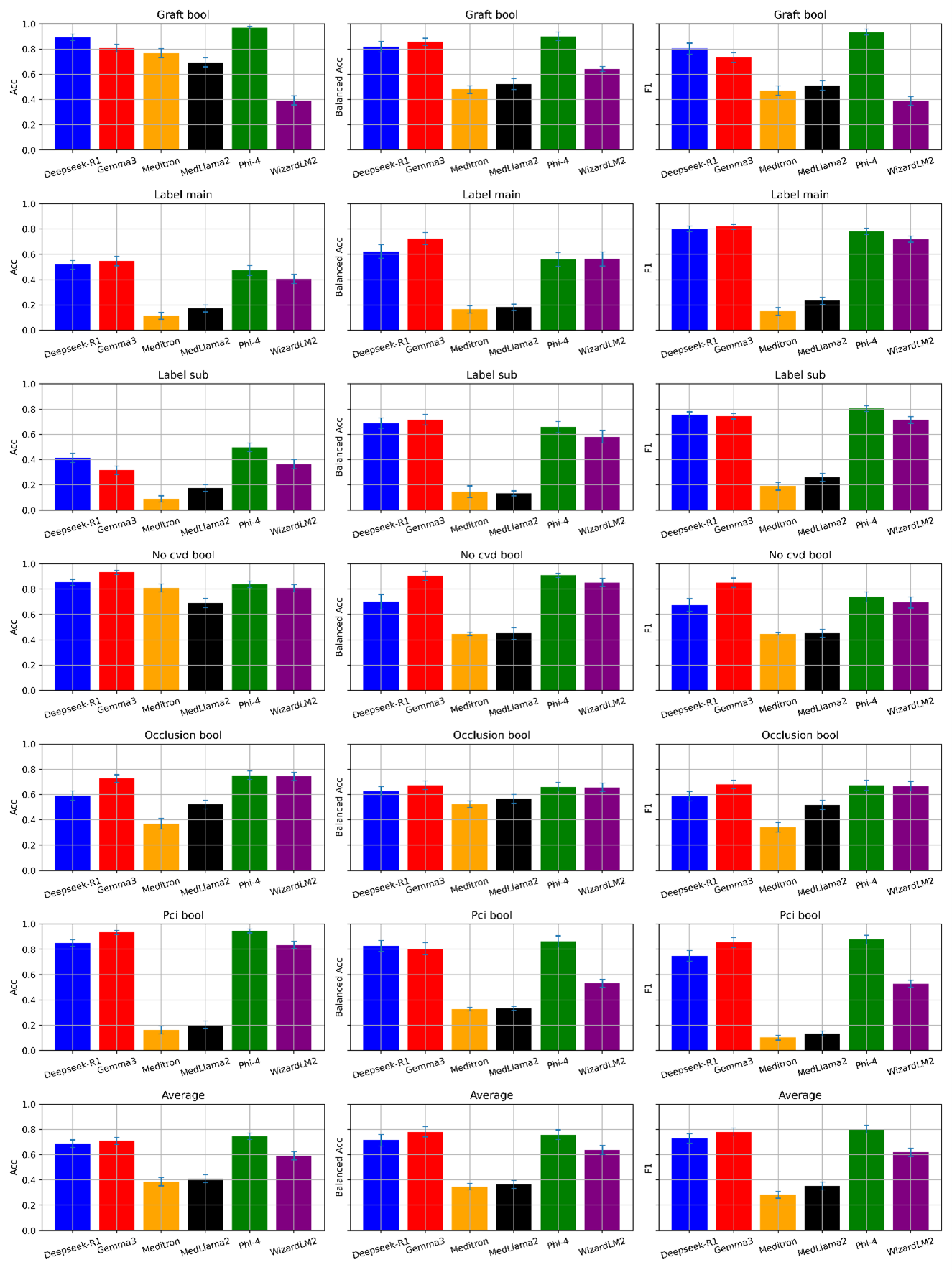

Supplement: ztaf127_Supplementary_Data [file ztaf127_supplementary_data.zip › SubFig10_LLM.jpg.png]

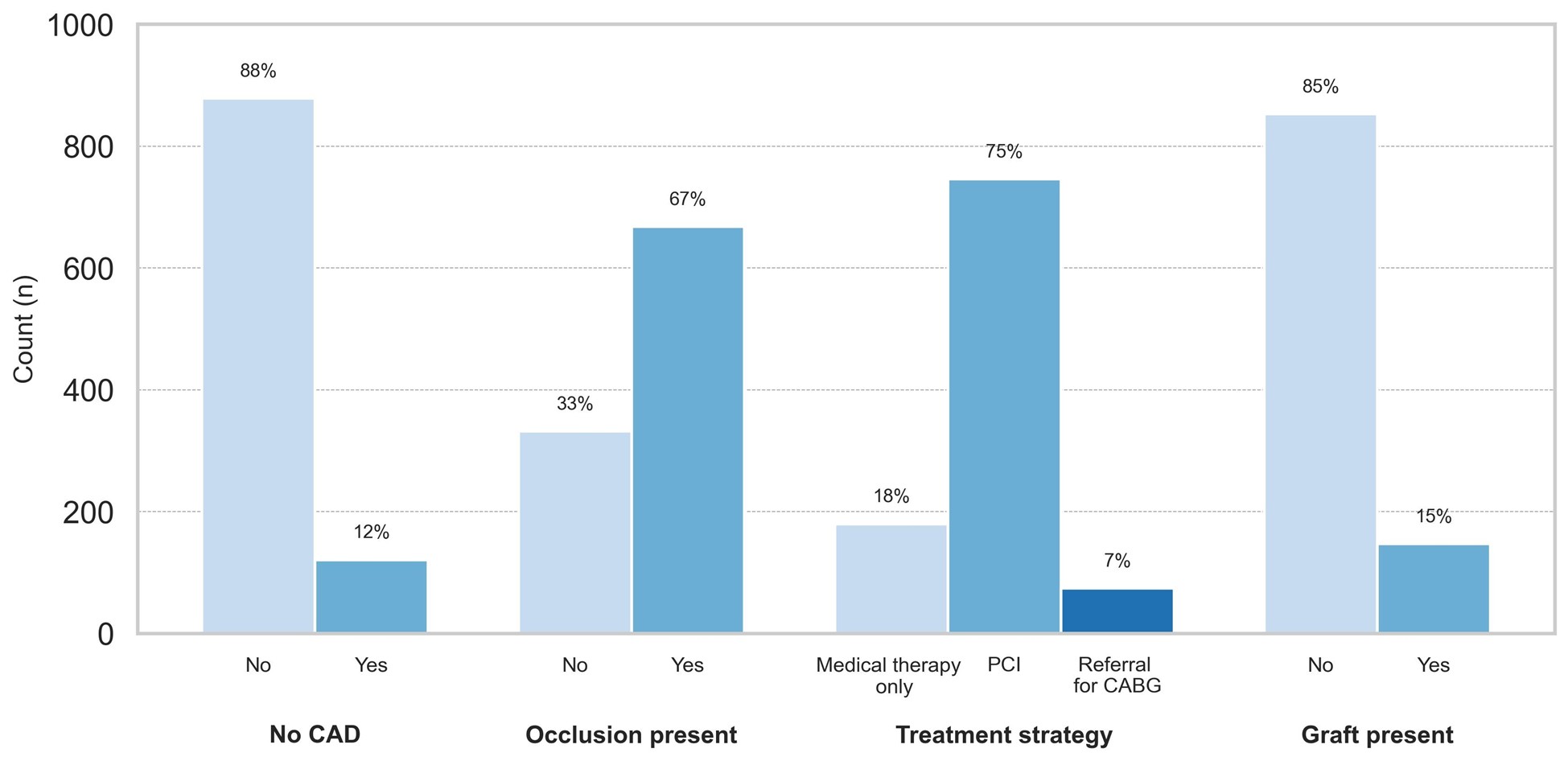

Supplement: ztaf127_Supplementary_Data [file ztaf127_supplementary_data.zip › SubFig1_LLM.jpg]

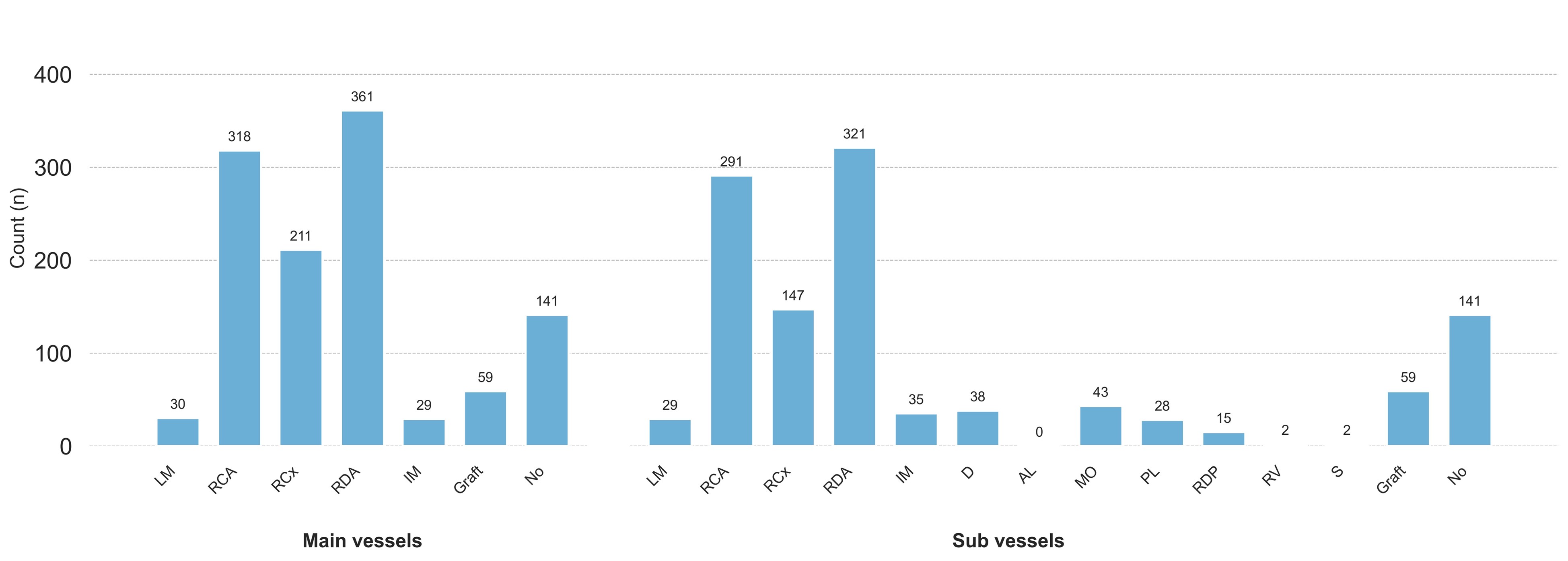

Supplement: ztaf127_Supplementary_Data [file ztaf127_supplementary_data.zip › SubFig2_LLM.jpg]

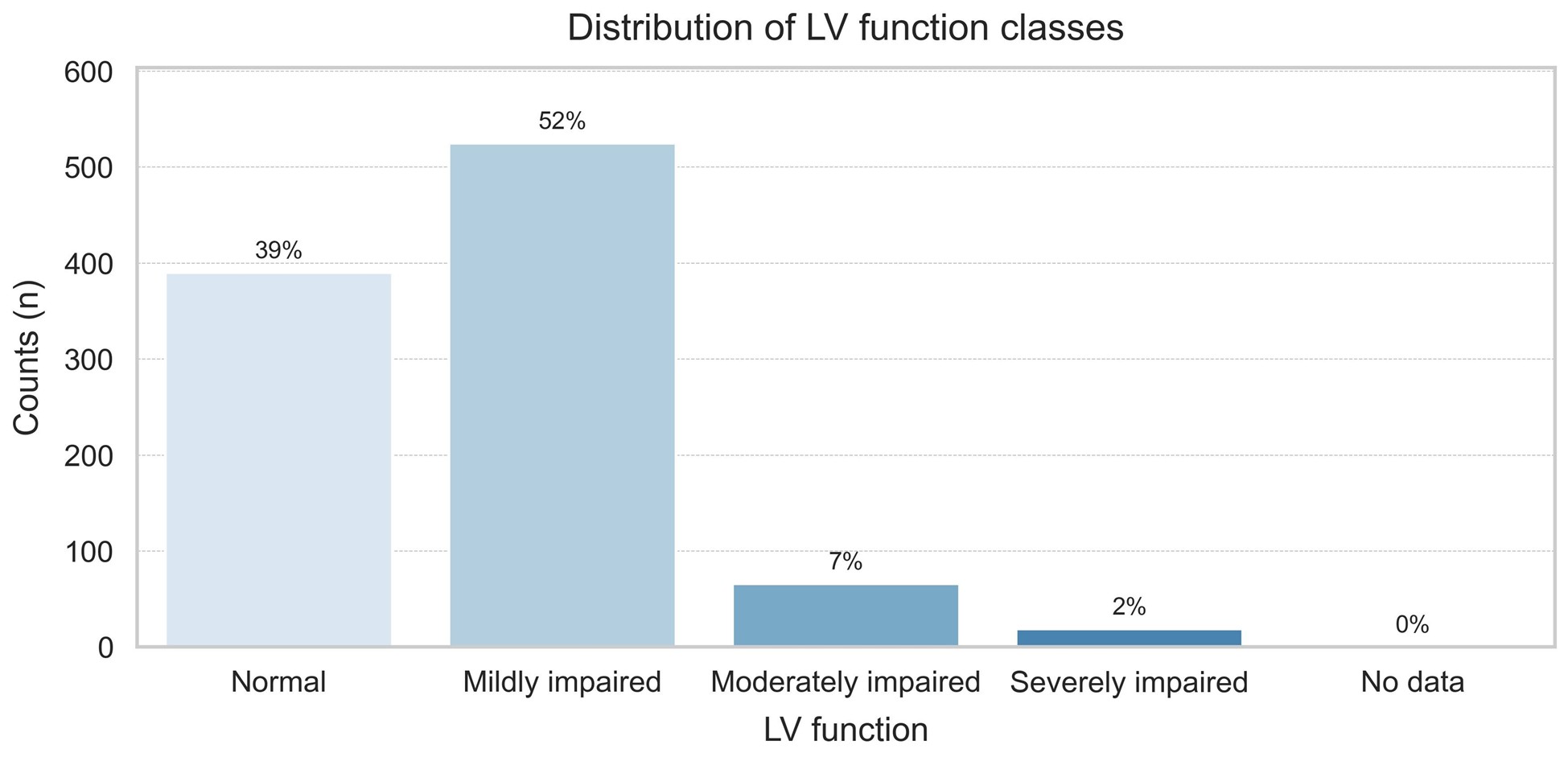

Supplement: ztaf127_Supplementary_Data [file ztaf127_supplementary_data.zip › SubFig3_LLM.jpg]

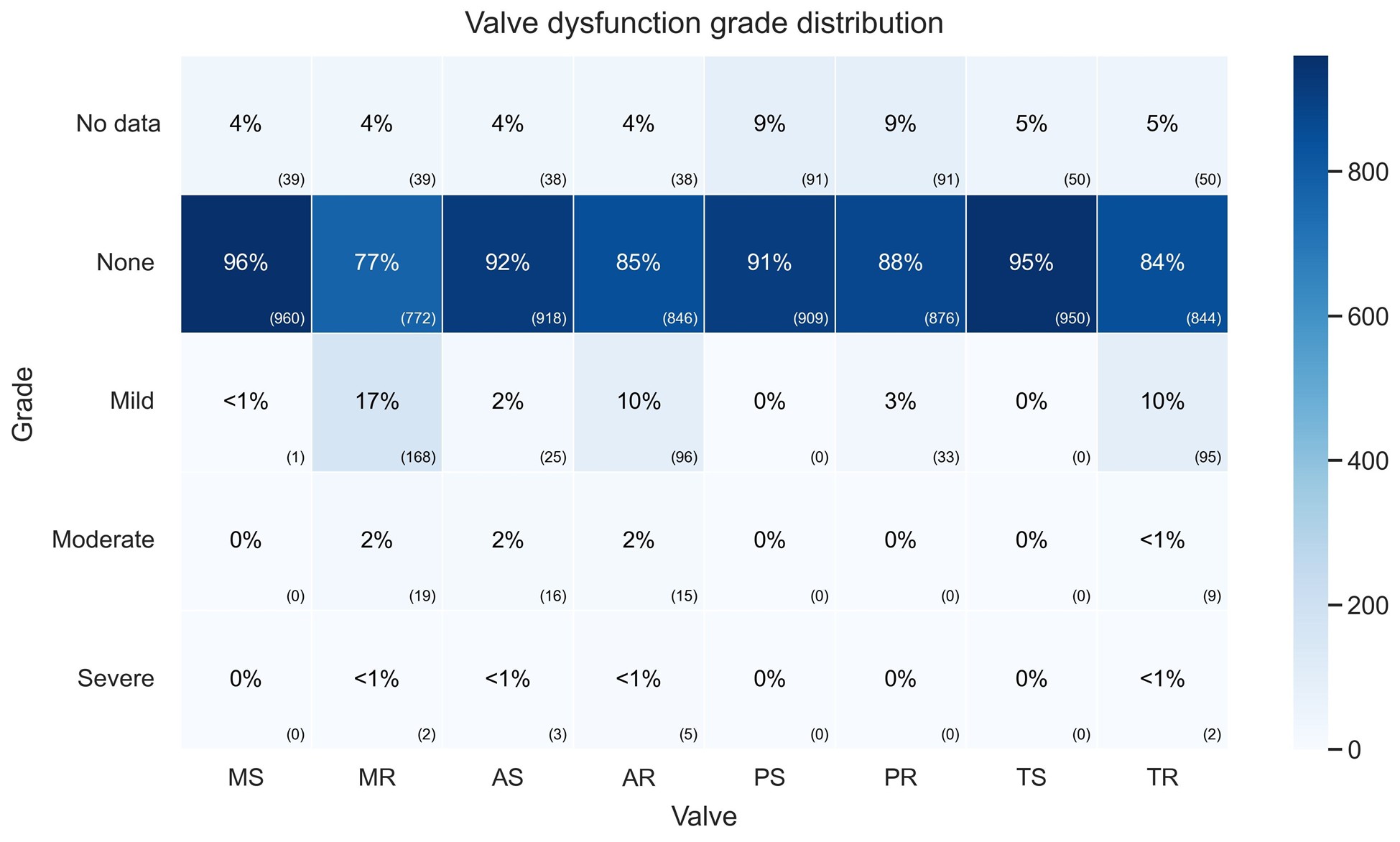

Supplement: ztaf127_Supplementary_Data [file ztaf127_supplementary_data.zip › SubFig4_LLM.jpg]

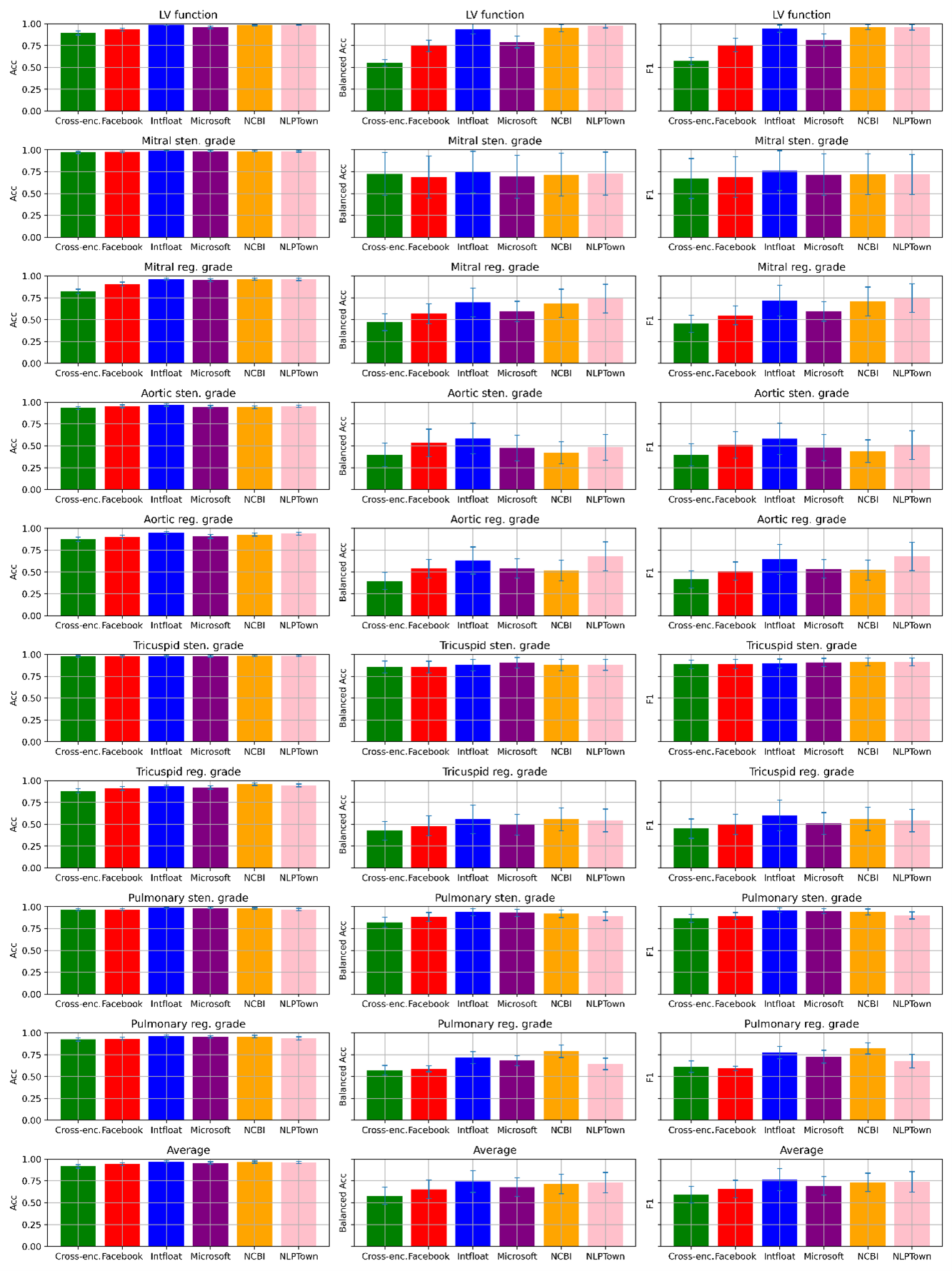

Supplement: ztaf127_Supplementary_Data [file ztaf127_supplementary_data.zip › SubFig5_LLM.png]

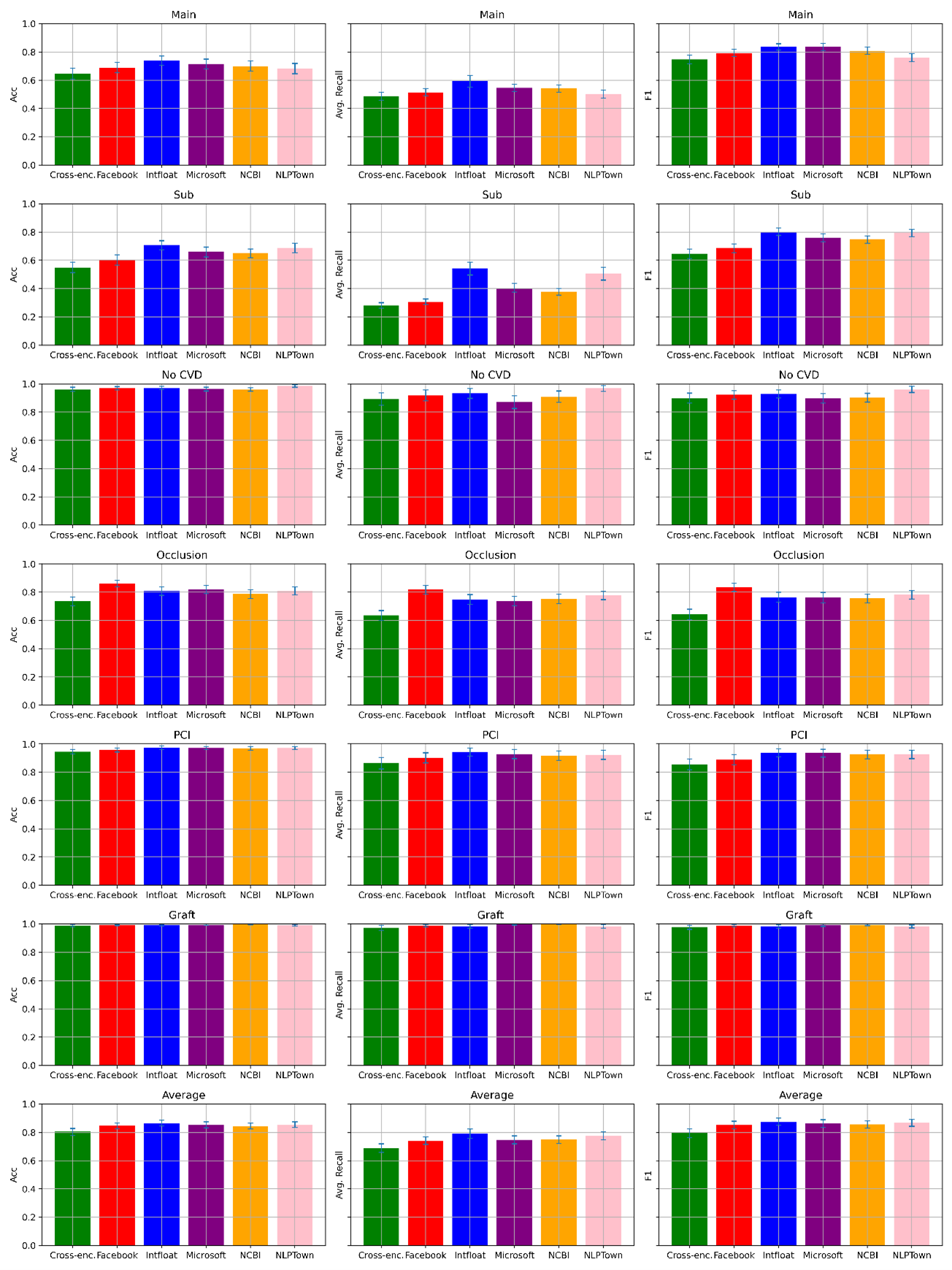

Supplement: ztaf127_Supplementary_Data [file ztaf127_supplementary_data.zip › SubFig6_LLM.png]
